# Anti-Contamination Strategies for Yeast Fermentations

**DOI:** 10.3390/microorganisms8020274

**Published:** 2020-02-18

**Authors:** Seung-Oh Seo, Sung-Kyun Park, Suk-Chae Jung, Choong-Min Ryu, Jun-Seob Kim

**Affiliations:** 1 Department of Food Science and Nutrition, The Catholic University of Korea, Bucheon 14662, Korea; drsos@catholic.ac.kr; 2 Infectious Disease Research Center, Korea Research Institute of Bioscience & Biotechnology (KRIBB), 125 Gwahak-ro, Daejeon 34141, Korea; skpark@kribb.re.kr (S.-K.P.); cmryu@kribb.re.kr (C.-M.R.); 3 Sempio Fermentation Research Center, Sempio Foods Company, 183 Osongsaengmyeong 4-ro, Cheongju 28156, Chungcheongbuk-do, Korea

**Keywords:** yeast fermentation, microbial contamination, anti-contamination strategy

## Abstract

Yeasts are very useful microorganisms that are used in many industrial fermentation processes such as food and alcohol production. Microbial contamination of such processes is inevitable, since most of the fermentation substrates are not sterile. Contamination can cause a reduction of the final product concentration and render industrial yeast strains unable to be reused. Alternative approaches to controlling contamination, including the use of antibiotics, have been developed and proposed as solutions. However, more efficient and industry-friendly approaches are needed for use in industrial applications. This review covers: (i) general information about industrial uses of yeast fermentation, (ii) microbial contamination and its effects on yeast fermentation, and (iii) currently used and suggested approaches/strategies for controlling microbial contamination at the industrial and/or laboratory scale.

## 1. Introduction

Yeasts may have first been used to produce fermented beverages by being accidentally introduced to sugar-containing liquids [1]. Yeast cell morphology was initially reported in the 17th century and conceptual clarity regarding yeast fermentation was brought about by scientists such as Louis Pasteur in the 19th century. Subsequently, yeasts have been used on an industrial scale to produce fermented beverages [2]. In general, yeasts convert sugar into alcohol (ethanol) and carbon dioxide, in a process called fermentation. However, the alcohol produced during fermentation threatens yeast survival, and yeasts have been developed that tolerate different alcohol concentrations in different products [3]. The yeast used in winemaking can tolerate 14%–18% alcohol, beer yeast 8%–12%, and baker’s yeast <8%.

### 1.1. Wine, Beer, and Bread

Wine is a type of yeast-fermented fruit beverage [4] and winemakers traditionally primarily used wild yeast of the genera *Candida*, *Pichia*, or *Zygosaccharomyces*, as well as yeasts from the family Metschnikowiaceae [5,6]. Because each of these wild yeasts produces their own unique flavors, they can produce high-quality wines. However, these yeasts are not suitable for industrial purposes because of their unpredictable behavior [7]. Therefore, industrial winemakers generally use *Saccharomyces cerevisiae*, a cultured yeast. Several hundred *Saccharomyces* strains (including hybrids inside genus) are used in winemaking [7]. Beer, which is produced by fermenting grains with yeast, is one of the most widely consumed commercial beverages. For beer making, cost-effective yeasts including *Saccharomyces cerevisiae* or *Saccharomyces pastorianus* (also known as *S. carlsbergensis*) are commonly used in the fermentation process [8]. In addition, non-*Saccharomyces* yeasts including *Brettanomyces* spp. are used to produce spicy and smoky flavored beers, while some strains of *Candida* and *Pichia*, which oxidize ethanol to acetaldehyde to provide a distinctive taste, are also commercially used [9]. The most commonly used baker’s yeasts for breadmaking are *S. cerevisiae* strains that have been selected for good properties such as robustness, large cell size, and high growth rate according to the needs of bakers [10]. Baker’s yeast ferments sugars in flour to produce alcohol and carbon dioxide, the latter serving as a leavening agent during breadmaking [10].

### 1.2. Biofuels

Climate change, one of the major threats to humans, stems from the excessive use of fossil fuels that, when combusted, produce greenhouse gases including carbon dioxide. Therefore, it is essential to replace fossil fuels with eco-friendly fuels. Current efforts are aimed at developing biofuels through the fermentation of renewable biomass by microorganisms [11]. Among the microorganisms used in such fermentation, yeasts are frequently used for alcohol production. *S. cerevisiae* is the most commonly used yeast for the first generation of bioethanol production from sugarcane juice or corn starch as a substrate [12]. For the second generation of bioethanol production using lignocellulosic biomass such as plants, the host strain of *S. cerevisiae* has been engineered to increase the consumption of sugars from the cellulosic hydrolysates [13]. Furthermore, yeast can be metabolically engineered to increase ethanol yield and produce higher alcohols other than ethanol. For example, *S. cerevisiae* ATCC 20602 has been genetically engineered to produce ethanol by utilizing glucose without oxygen [14], and *S. cerevisiae* A267T/E568K has been modified to harbor an *adh1* mutation to enhance n-butanol production [15]. The metabolically engineered strain *S. cerevisiae* YPH 499 has been primarily developed for isobutanol production, a next-generation biofuel that is expected to be used as a high-quality alternative to gasoline. This strain has been modified to enhance isobutanol production by upregulating several enzymes including pyruvate carboxylase while inhibiting the pyruvate dehydrogenase complex that would otherwise interfere with isobutanol synthesis [16]. Additionally, several key enzymes for the isobutanol biosynthesis pathway were overexpressed in the cytosol of *S. cerevisiae* to enhance isobutanol production in yeast [17]. One of the industrially important higher alcohols, 2,3-butanediol, has also been highly overproduced in the metabolically engineered *S. cerevisiae* through a redirection of carbon flux from ethanol to 2,3-butanediol [18]. Isopropanol, which has various industrial and domestic applications, can be produced by *Candida utilis* engineered to maximize the efficiency of isopropanol production by overexpressing acetyl-CoA-synthetase and acetyl-CoA-acetyltransferase [19]. Furthermore, *Kluyveromyces lactis* and *P. pastoris* produce isoamyl alcohol by consuming glucose and yeast extract [20].

### 1.3. Protein Production

Recombinant proteins constitute a multi-million-dollar market [5]. In particular, the production of therapeutic proteins has yielded significant advancements in the development of molecular medicines [21]. Among several expression systems synthesizing biopharmaceutical proteins for human diseases, yeast cells are the most useful system. Yeasts are unicellular and so are amenable to large-scale production, can be grown rapidly, and their genomes can be easily manipulated. Furthermore, yeast cells are eukaryotic hosts and have secretory pathways enabling protein folding or post-translational modifications leading to the production of functional proteins in mammal systems [22]. In the production of recombinant biopharmaceuticals, the world’s most marketed product is insulin or its analogs, which are administered to individuals with diabetes [23]. *S. cerevisiae* is one of the most widely used expression hosts for large-scale production of these proteins. Besides diabetes treatment, *S. cerevisiae* is also used to produce vaccines against various infectious diseases [22]. For example, recombinant protein vaccines including Ambirix, Pediarix, or Twinrix for hepatitis A or B [24] and Infanrix for diphtheria, tetanus, and pertussis are representative drugs manufactured using *S. cerevisiae* as the expression system [25]. As a consequence of the ease of genome manipulation of *S. cerevisiae*, various strains have been constructed to provide the optimum host for different applications. *S. cerevisiae* SIC, generated using the pSynInsCPOT plasmid for increased insulin production when compared with the conventional strain, is a representative example [26,27]. Beyond *S. cerevisiae*, *P. pastoris* has been used to produce the human p53 tumor suppressor protein [28], and *Schizosaccharomyces pombe* has been widely used to produce hepatitis vaccines [24].

## 2. Microbial Contamination during Yeast Fermentation

### 2.1. Wine Fermentation

In the wine industry, spoilage has been mainly caused by bacteria such as lactic acid bacteria (LAB). However, wine spoilage by unwanted bacteria is currently very rare, owing to the development of improved production technologies and good manufacturing practice. Conversely, in present, many wine spoilage incidents are caused by contamination with yeasts such as *Dekkera* spp. [29]. Usually, the grapes and grape juice are not sterilized prior to wine fermentation. Wild yeasts are located on the surfaces of the grapes and grape juice containers, which microbial cells can adhere to and colonize [30]. Consequently, various yeasts such as *Dekkera bruxellensis*, *D. anomala*, *D. naardenensis*, *D. custersiana, D. nanus, Candida halophila*, *C. cantarelli*, *Meyerozyma guilliermondii*, and *K. lactis* have been identified in damaged grapes and the grape juice made from them [30,31]. Analysis of these yeasts showed that *M. guilliermondii* converted p-coumaric acid to 4-ethylphenol in a manner similar to *D. bruxellensis* [31]. However, after wine fermentation, *M. guilliermondii* could not be recovered whereas *D. bruxellensis* could. Because of this, *D. bruxellensis* is considered to be the only causative agent of phenolic off-odor and off-flavor production in wine fermentations [31,32]. *D. bruxellensis* can metabolize free-form hydroxycinnamic acids (p-coumaric acid, caffeic acid, and ferulic acid) [33]. P-coumaric acid is converted to 4-ethylphenol, a volatile phenol, and unpleasant aromas occur as the concentration of 4-ethylphenol increases [34,35]. In the overall wine production process, *D. bruxellensis* can grow on damaged grapes in the vineyard to contaminate the grape juice, thus causing the production of volatile phenols (especially 4-ethylphenol) during fermentation (Table 1).

### 2.2. The Brewing Industry

The brewing process of beer may be exposed to microbial contaminants from a variety of sources. Even though the primary sources of contaminants are the raw materials and the brewhouse vessels, additional contaminants can be introduced during the bottling, canning, and kegging processes. Mostly, LAB, acetic acid bacteria, and obligately anaerobic bacteria have been identified in contaminated beers [36,37]. Among them, *Lactobacillus brevis* accounts for more than half of the spoilage bacteria found [38] (Table 1).

Most bacteria exposed to bitter acids released from hops added into the brewing process have growth inhibition due to the toxicity [37,39,40,41]. However, *L. brevis* expresses HorC, which results in resistance to the hop bitter acids and thereby circumvents growth inhibition [37,42,43]. *L. brevis* causes spoilage of beer, producing a variety of off flavors and aromas, as well as increasing the turbidity of the final product [38,44,45,46].

Raw materials for brewing such as barley, malt, hops, and adjuncts contaminated with fungal mycotoxins may contaminate final beer products. Mycotoxins that affect health include aflatoxins (AF), ochratoxin A (OTA), patulin (PAT), trichothecenes (deoxynivalenol DON, nivalenol NIV, HT-2 toxin, and T-2 toxin), zearalenone (ZEN), and fumonisins (FUM) [47]. These mycotoxins are mainly produced by the fungal genera *Aspergillus* (AF, OTA, and PAT), *Penicillium* (OTA and PAT), and *Fusarium* (DON, NIV, HT-2, T-2, and ZEN). Mycotoxin contamination occurs at different steps of the brewing process. Some of the mycotoxins can be transferred from raw materials to malt and beer as a consequence of the high thermal stability of AF, ZEN, and DON, and the water solubility of DON and FUM [48].

Barley can be affected by a plant disease called Fusarium head blight, which is caused by the *Fusarium* species [49]. This disease damages the brewing industry through a negative impact on the barley germination rate and mycotoxin contamination [50,51]. High-quality barley can also be infected during malting. If the *Fusarium* is introduced during the steeping step of malting, the fungal biomass increases during germination and decreases during the kilning step. In this case, the mycotoxin DON is detected at the beginning of germination and disappears at the kilning step. However, if the malting process is started with infected barley, there is a continuous elevated *Fusarium* biomass from the steeping to kilning steps, as well as a high DON concentration [52]. Moreover, alcohol dehydrogenase is inhibited by some of the mycotoxins from contaminated barley, thus increasing the concentration of acetaldehyde and other volatile compounds during alcohol fermentation [53,54].

### 2.3. Fuel Ethanol Fermentation

Microbial contaminants affecting fuel ethanol fermentation can be divided into yeasts and bacteria. In the case of yeasts, *Dekkera*, *Schizosaccharomyces*, and C*andida* are considered as the main contaminants. In the case of bacteria, LAB are the most common contaminants and cause decreased ethanol production through competition with the yeast for available sugars and nutrients, and the accumulation of by-products such as lactic acid and acetic acid [55,56,57].

The fuel ethanol fermentation industry uses a fed-batch or continuous fermentation system [55]. These systems use non-sterilized cane juice and/or molasses as a carbon source, and recycled yeast obtained by centrifugation as an inoculum. As a result, contamination of bacteria and non-*S. cerevisiae* yeasts can occur in fermentation. In cases where the yield of ethanol was significantly reduced during fermentation, a relatively high concentration of contaminating non-*S. cerevisiae* yeast was observed. *D. bruxellensis* is known as the main contaminant yeast in Brazil [58]. Additionally, *Dekkera* spp. have been reported as important contaminant non-*S. cerevisiae* yeasts in ethanol fermentation processes in the USA, Canada, and Europe [59,60] (Table 1).

Contamination by *D. bruxellensis* has resulted in lower productivity, lower ethanol yield, and high residual sugar concentration [58,61]. The reason for the decrease in ethanol production by contamination of *D. bruxellensis* is unclear. The growth rate of *D. bruxellensis* is much lower than that of *S. cerevisiae* in fully supplemented and freshly inoculated natural media in laboratory batch fermentations, either in aerobic or anaerobic conditions [60]. However, *D. bruxellensis* grows faster than *S. cerevisiae* in industrial continuous fermentations [58]. A concentration of acetic acid above 0.45% (w/v) strongly inhibits the growth of *S. cerevisiae,* while *D. bruxellensis* has tolerance against acetic acid at this concentration and higher [59]. *D. bruxellensis* produces more acetic acid than *S. cerevisiae* under various aeration conditions. Notably, the production of acetic acid by *D. bruxellensis* in semi-anaerobic conditions is three times higher than that of *S. cerevisiae* [60]. As a result, *S. cerevisiae* cannot grow well, but *D. bruxellensis* can grow and become dominant [59,60]. Bassi et al. reported that, when *S. cerevisiae* and *D. bruxellensis* were co-cultured in molasses media, the growth rate of *D. bruxellensis* was faster than that of *S. cerevisiae,* with an increasing number of fermentation cycles. As a result of co-culture in molasses media, residual glycerol and acetic acid concentrations were increased by 15% and 70%, respectively, and ethanol yield was reduced by 20%, compared with inoculating *S. cerevisiae* alone [62]. In addition, when there was contamination by *D. bruxellensis* together with *L. fermentum*, the ethanol yield was further reduced by 90% with decreased viability of *S. cerevisiae* [62]. Recently, one study found that the *D. bruxellensis* strain has the capacity of producing ethanol at a similar yield as *S. cerevisiae* under strict anaerobic conditions, when nitrate is used as a nitrogen source [63].

*L. fermentum* produces lactate, acetate, and mannitol from the mixture of glucose and fructose in sugarcane juice. The acidic products cause a decrease in sugar consumption, growth inhibition of *S. cerevisiae*, and a decrease in ethanol production [64]. If *S. cerevisiae* is contaminated by *L. fermentum,* flocculation (co-aggregation) of *S. cerevisiae* with *L. fermentum* can occur [65,66,67]. *S. cerevisiae*–*L. fermentum* flocculant produces organic acids at a higher level than axenic *S. cerevisiae* [68]. According to the transcriptional analysis, the number of down-regulated genes of *S. cerevisiae* in the co-culture of the *S. cerevisiae*–*L. fermentum* flocculant was four times higher than in axenic cultures. This result suggested that the concentrations of organic acids cause down-regulation of genes in *S. cerevisiae*, thereby reducing cell growth rates, sugar consumption, and ethanol yield [68].

## 3. Anti-Bacterial Contamination Strategies in the Laboratory and Industry

### 3.1. Chemical Agents

To combat unwanted bacterial contamination in yeast fermentation, antimicrobial chemical agents can be used. Some chemicals, including acids, ammonia, and hydrogen peroxide, which are toxic to the bacterial cells, have been tested and employed for controlling bacterial infection in yeast fermentations. One of the treatments commonly used in the bioethanol industry is the acid-washing of yeast cells with diluted sulfuric acid before and after fermentation, to reduce bacterial contaminants [69]. Sulfuric acid solution with 5% ethanol (pH 2.0) can be more effective than using sulfuric acid solution alone in the removal of total cell growth of *L. fermentum* contaminant after the first fermentation cycle [70]. Performic acid (provided by DesinFix^TM^ 135, Kemira, Finland) can significantly reduce numbers of LAB including *L. fermentum*, *Lactobacillus paracasei*, *Lactobacillus plantarum*, and *Leuconostoc mesenteroides* without affecting yeast growth and the fermentation performance, by adding a small amount of the solution (5 to 60 mg/L) to yeast cream on each day of batch fermentation [71]. However, acid-washing and multiple recycling may reduce the viability of yeast cells resulting in decreased efficiency. Other than acids, ammonia has been used for the treatment of substrate for ethanol fermentation to decrease bacterial contamination from the substrate [72] and to hydrolyze lignocellulosic biomass [73]. Yeast fermentation using corn grains disinfected by weak ammonia solution (0.5% to 1.5% *w*/*v*) resulted in a decrease of growth of lactic acid bacteria and molds and an increase of ethanol production when compared with using non-disinfected corn grains [72]. Urea hydrogen peroxide (30 to 32 mmol/L) was also effective for reducing the numbers of multiple LAB contaminants including *L. plantarum*, *L. paracasei*, *L. rhamnosus*, and *L. fermentum* in wheat mash without altering the growth of yeast during ethanol fermentation for 36 h [74]. Hydrogen peroxide at concentrations of 1 to 10 mmol/L and sulfite at concentrations of 100 to 400 mg/L effectively reduced selected LAB strains such as *L. casei* and *L. fermentum* during cell-recycled ethanol fermentations [75]. One of the well-known biocides used in water treatment, chlorine dioxide (ClO_2_), has been tested to control bacterial contamination by LAB contaminants (*L. plantarum*, *L. fermentum*, and *L. mesenteroides*) and *Bacillus subtilis* in alcohol fermentation [76]. Chlorine dioxide in the range of 10–200 ppm successfully inhibited the growth of the bacterial contaminants, but a concentration higher than 50 ppm also affected the growth of industrial yeasts [76]. One study reported that the use of chemical 3,4,4,’-trichlorocarbanilide (TCC) at a concentration of 0.075 g/L resulted in excellent control of bacterial growth (*L. fermentum*) for multiple fermentation cycles in fed-batch ethanol fermentation when used with sodium dodecyl sulfate [77]. In winemaking, sulfur dioxide (SO_2_) has been commonly used as an antimicrobial agent to inhibit the development of unwanted bacteria, mold, and spoilage yeasts during fermentation and preservation [78,79]. However, it is often problematic due to allergic reactions in some consumers caused by the sulfites that result from the SO_2_ being added to the wine [80,81]. Although chemical treatment can effectively control bacterial contamination and growth in yeast fermentation, these chemicals can be detrimental to the yeast and pose environmental risks because of their general toxicity. Therefore, more specific methods for contamination control, such as the use of antibiotics, have been considered.

### 3.2. Antibiotics

The use of antibiotics is an effective method to selectively kill bacterial contaminants in yeast fermentations. The bioethanol industry often uses various antibiotics such as penicillin G, tetracycline, virginiamycin, or a cocktail of these antibiotics against *Lactobacillus* spp. and other bacterial species in fermentations at a range of concentrations from 0.1 mg/L to 20 mg/L [82,83,84,85]. Among the antibiotics, virginiamycin has been widely considered for yeast fermentation since it shows activity against lactobacilli at lower pH values than other antibiotics [86]. However, there are two major problems associated with using antibiotics. These are residual antibiotics in the fermentation broth and the emergence of drug-resistant strains. In addition, the quantities and costs of antibiotics used in large-scale fermentation are significant. In the case of yeast fermentation for food production, these issues are more problematic because of strict food-safety regulations [87]. To avoid these concerns regarding widespread antibiotic resistance and residues, better strategies for managing bacterial contamination using alternatives to antibiotics have been considered for food fermentation, despite the effectiveness of antibiotics.

### 3.3. Natural Antimicrobial Compounds and Bacteriocins

Instead of using antibiotics, biological antimicrobial agents including natural products and bacteriocins can be used to control bacterial contamination [88]. Natural antimicrobial compounds are derived from different natural sources such as animals, plants, and microorganisms, and have been used for the treatment of human infections as well as the prevention of food spoilage [89,90,91]. One of the traditional examples is the addition of hops to the brewing process [92]. Currently, hop acid products extracted from hops are commercially available and applied in the production of bioethanol from distillers’ grains at concentrations of 10 to 50 mg/L [93]. As hops provide the unique taste and bitterness of beer products, other natural compounds may provide the similar benefits to the brewing process. Since herbs and spices such as cinnamon, cassia, garlic, basil, ginger, and mustard possess antibacterial activities as well as unique flavors [94], herb extracts may be utilized as natural substitutes for antibiotics and flavoring agents in beer production. A similar antibacterial activity can also be found in fungi belonging to the family Basidiomycota, i.e., mushrooms [95,96]. They produce many bioactive compounds including flavonoids, alkaloids, phenolics, and terpenoids that can be used as ingredients for functional foods, medicines, and cosmetics, as well as acting as food preservatives [97,98]. Thus, edible mushroom extracts can be utilized in the brewing process to prevent bacterial contamination during fermentation, and offer a function to alcoholic beverage production.

One study found that chitosan extracted from the shells of crabs and shrimps can decrease the viability of LAB contaminants including *L. plantarum* and *Pediococcus* spp. isolated from beer fermentation at concentrations of 0.1 to 1 g/L [99]. The use of chitosan in beer production may help to reduce food waste by increasing the use of, and hence demand for, seafood shell waste. Another interesting approach is the use of lignocellulosic hydrolysates from plant biomass as natural antibacterial agents, which might replace antibiotics in bioethanol fermentation [100]. For the second generation of bioethanol production, pretreated plant biomass and its hydrolysate could be utilized as both substrates and antimicrobial agents. One of the drawbacks of these natural compounds is that some phytochemicals show a negative effect on yeast viability. Furthermore, like other antibiotics, natural antimicrobial compounds are known to be subject to microbial resistance [92,101].

Another group of natural antimicrobial agents with high specificity are the peptides known as bacteriocins. These are secreted mainly from LAB to compete with other LAB of the same species or other genera [102]. Considering that the main contaminants in ethanol fermenters are LAB [103], a cocktail of various bacteriocins may effectively control the LAB contaminants in yeast fermentations. One good example of a bacteriocin is nisin, a molecule that has been well studied for food applications [104]. Nisin has been tested for controlling bacterial contamination in wine and beer production [105,106]. However, bacteriocins like nisin are expensive to produce, and this limits their use to small-scale brewing process studies [104].

### 3.4. Bacteriophages and Endolysins

Unlike bacteriocins, the use of bacteriophages as antibacterial agents can be cost effective, since they infect and multiply in bacteria starting from a small dosage. Since bacteriophages can lyse bacteria without a negative effect on human or animal cells, they have received increased attention in clinical therapy [107]. Furthermore, bacteriophages have no impact on the growth of yeast and have been considered for the control of bacterial contamination in bioethanol fermentation [108]. However, the main drawback of using bacteriophages is the development of resistance in bacteria against bacteriophage infection [109]. The risk of emergence of bacterial resistance to bacteriophages limits the application of bacteriophages as antimicrobial agents. However, bacteriophage-encoded lytic enzymes that allow the bacteriophages to lyse bacterial cells can be used for the control of bacterial contamination as an alternative to bacteriophages. For example, the endolysin LysA2 from a *Lactobacillus casei* bacteriophage can reduce the growth of a wide spectrum of LAB [110]. The antimicrobial potential of LysA2 has been tested and used for the reduction of contamination problems in fuel ethanol production [108,111]. Like bacteriocins, the production cost of the endolysin protein is significant. However, if the endolysin is produced by yeast, this cost could be reduced [112].

### 3.5. Other Methods

Ozone treatment of substrates, such as corn mash, for ethanol production results in the reduction of lactic acid production by LAB contaminants [113]. Furthermore, bacterial contaminants can be directly removed from the bioethanol fermentation system by using membrane bioreactors [114]. This system allows physical selective separation of yeast and contaminating bacteria in an immersed membrane bioreactor and increases final ethanol yields. Another approach to reducing contamination is to use native killer yeasts that are known to inhibit the growth of bacteria and other yeast species [115]. Recently, metabolic engineering has been applied to allow the yeast to outcompete contaminating organisms by utilizing rare compounds as sources of nutrients [116]. This system used a customized fermentation environment to limit the growth of other wild contaminants through a lack of nutrients such as nitrogen and phosphorus. Then, essential growth nutrients were provided through low-cost xenobiotic or rare chemicals to allow the growth of engineered yeast capable of using them. This strategy may significantly reduce the cost of industrial fermentations by avoiding the need for feedstock refining, sterilization, antibiotics, or other antimicrobial agents for the prevention of microbial contamination.

## 4. Conclusions

**Figure 1:**
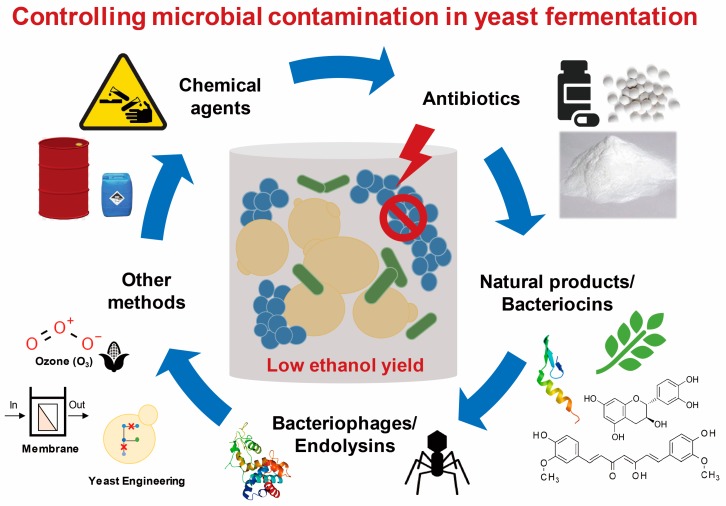
Strategies for controlling microbial contamination in yeast fermentation reviewed in this study. This figure describes the strategies for preventing unwanted bacteria and yeast contamination in alcoholic fermentation by using (**i**) chemical agents such as acids and bases, (**ii**) antibiotics, (**iii**) natural antimicrobial compounds and bacteriocins, (**iv**) bacteriophages and their endolysins, and (**v**) other methods such as disinfection of substrate by ozone treatment, use of membrane bioreactor, and engineering of yeast as a robust host.

Yeasts are the most common microorganisms used for industrial applications and play important roles in the production of food, biofuel, and various bio-products. Fermentation by yeast mediates the conversion of various carbon sources to not only ethanol but also other beneficial products. Since complete sterilization of feedstock is not possible, substrates for industrial yeast fermentations are contaminated by various bacteria. Such contaminants inhibit the growth of yeasts, reduce the efficiency of fermentation processes, and hence significantly reduce productivity. Various anti-bacterial decontamination strategies have been developed and applied in the industry (Figure 1), but it is still necessary to improve their efficacy and reduce unwanted side-effects. Additionally, more selective methods preventing contamination of non-*S. cerevisiae* yeasts in the alcoholic fermentation by *S. cerevisiae* need to be developed. In investigating the transfer of anti-contamination strategies from the laboratory to industry, many issues have arisen such as environmental safety and side-effects on the inoculum yeast strain. Given the importance of yeast fermentation to human society, safer and more efficient strategies need to be further developed in a sustainable way.

## Figures and Tables

**Table 1:** List of major contaminants in wine, brewing, and fuel ethanol fermentation industries.

Fermentations	Major Contaminants			Contaminant’s Original Source
	Yeast	Bacteria	Fungi	
Wine fermentation	*D. bruxellensis*			grape
Brewing fermentation		*L. brevis*	*Fusarium* species	barley, malt, hops, or adjuncts
Fuel-ethanol fermentation	*D. bruxellensis*	*L. fermentum*		cane juice and molasses
